# Case Report: Incidental diagnosis of cystic fibrosis via whole genome sequencing alters HSCT planning in a child with cerebral X-linked adrenoleukodystrophy

**DOI:** 10.3389/fped.2025.1650645

**Published:** 2025-08-11

**Authors:** Jann Adriel Chua Sy, Poh Lin Tan, Jeremy Bingyuan Lin, Stacey Kiat-Hong Tay, Hui-Lin Chin

**Affiliations:** ^1^ Khoo Teck Puat – National University Children’s Medical Institute, National University Health System, Singapore, Singapore; ^2^ Department of Paediatrics, Yong Loo Lin School of Medicine, National University of Singapore, Singapore, Singapore

**Keywords:** cerebral X-linked adrenoleukodystrophy, haematopoietic stem cell transplantation, cystic fibrosis, whole genome sequencing, incidental findings

## Abstract

Cerebral X-linked adrenoleukodystrophy (cALD) is an X-linked peroxisomal disorder caused by pathogenic variation in the *ABCD1* gene, characterized by progressive central nervous system demyelination leading to severe neurocognitive decline, as well as concomitant adrenal insufficiency resulting from fatty acid accumulation. When instituted early, haematopoietic stem cell transplantation (HSCT) can halt neurological disease progression. Whole genome sequencing can clarify the diagnosis of cALD but may also reveal conditions with significant clinical implications, as in our case. To our knowledge, this is the first published case where an incidental finding of cystic fibrosis influenced pre-HSCT workup and subsequent management in a child with cALD. A 6-year-old boy presented with subacute neuroregression, manifesting as deteriorating cognition and speech, hyperactivity, clumsiness, and swallowing dysfunction. Brain MRI confirmed symmetrical demyelinating lesions consistent with cALD, and whole genome sequencing (WGS) identified a maternally-inherited pathogenic *ABCD1*(NM_000033.4):c.521A>G (p.Tyr174Cys) variant. WGS also incidentally identified compound heterozygosity for two pathogenic variants in *CFTR*, supporting an incidental cystic fibrosis (CF) diagnosis, clinically verified by an abnormal sweat test and radiological findings of subclinical bronchiectasis, as the child was asymptomatic. This influenced the HSCT conditioning regimen prescription and reinforced the need for enhanced infectious prophylaxis and vigilant respiratory support. This case highlights the potential of comprehensive genomic approaches to reveal previously undetected comorbidities. Integrating CF management into a cALD-directed HSCT protocol mitigated peri-transplant risks, demonstrating the value of multidisciplinary care when incidental diagnoses emerge.

## Introduction

Cerebral X-linked adrenoleukodystrophy (cALD) is a devastating neurodegenerative condition driven by pathogenic variants in the peroxisomal transporter gene *ABCD1*, leading to toxic accumulation of very long-chain fatty acids (1). Untreated patients often experience rapid neurologic decline, including white matter demyelination and adrenal insufficiency, and most would pass away within 3–4 years following presentation (2). Haematopoietic stem cell transplantation (HSCT) has proven efficacious in stabilizing or halting disease progression in early disease stages, characterised by low (<10) Loes score-based imaging and minimum major functional disabilities (3). Furthermore, lentiviral gene therapy using autologous stem cells is also emerging as an alternative strategy for treating cALD (4).

Although there is no standard for performing comprehensive genomic testing for patients undergoing HSCT for cALD, genetic analysis has become widely used to confirm the suspected cALD diagnosis. Genetic testing can be performed using single-gene sequencing and deletion/duplication analysis, gene panel testing, exome sequencing, or genome sequencing. Although the primary aim is to validate the presumed diagnosis, if comprehensive sequencing (exome or genome sequencing) is performed, incidental or secondary findings—particularly in actionable genes (5)—may significantly alter clinical management, as in our case. Among such genes is *CFTR*, responsible for cystic fibrosis (CF)—an autosomal recessive disorder characterised by chronic pulmonary infections and intestinal malabsorption (6). Incidental detection of *CFTR* pathogenic variants in a cALD patient who is undergoing HSCT raises unique challenges, including the selection of the optimum conditioning regimen (which can affect pulmonary function), supportive management during the period of profound cytopenia with a high risk for infection, and late effects follow up for pulmonary complications such as graft vs. host disease of the lung. Other incidental findings in paediatric HSCT patients that may be clinically relevant include those that necessitate modifying the intensity of conditioning, such as in Fanconi Anaemia or DNA-repair mismatch syndromes, which confer toxicity to chemotherapy and radiotherapy and may predispose to secondary malignancy later on (7).

We present a 6-year-old boy diagnosed with cALD who, through WGS, was also found to have compound heterozygous pathogenic *CFTR* variants. We discuss how this unexpected CF finding prompted the use of pulmonary prophylactic strategies, analysis of the ideal HSCT conditioning regimen, and pre- and post-transplant monitoring for pulmonary complications. This case report highlights the critical implications of incidental genomic discoveries in children with complex inherited diseases.

## Case description and diagnostic assessment

A 6-year-old Malay-Pakistani boy of non-consanguineous parents presented with a 3-month history of gradual neuroregression, manifesting as deteriorating cognition, hyperactivity, speech, clumsiness, and swallowing dysfunction. He was previously well, with no history of recurrent respiratory infections or chronic diarrhoea.

Physical examination revealed normal vital signs and growth. He had dermal and mucosal hyperpigmentation. Tone, power, and reflexes were normal. He had difficulties with fine motor tasks, and his speech was slurred and incomprehensible. Assessment of vision was otherwise normal.

**Figure 1:**
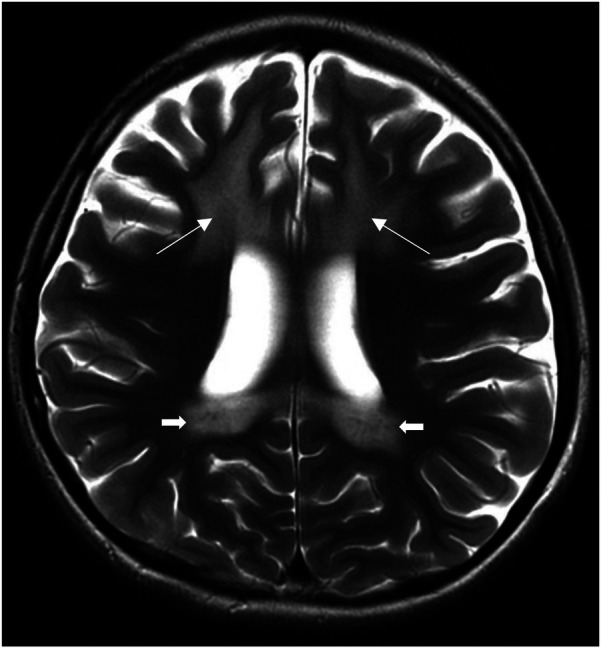
Axial T2-weighted MRI brain images at presentation showing symmetrical T2-weighted hyperintense signals of the splenium, internal capsule (arrows).

Magnetic resonance imaging (MRI) of the brain and cervical spine revealed symmetrical T2-weighted hyperintense signals involving the bilateral frontal periventricular and deep white matter, corticospinal tracts within the corona radiata, and posterior limb of the internal capsule, splenium of the corpus callosum, and cerebellar white matter (Figure 1) demonstrates representative T2-weighted hyperintense signals of the splenium and internal capsule.

The serum very long chain fatty acid profile showed high C26 levels, as well as C24:0-to-C22:0 and C26:0-to-C22:0 ratios, consistent with X-linked adrenoleukodystrophy. Synacthen testing confirmed primary adrenal insufficiency. Table 1 summarises the biochemical results consistent with X-linked cerebral ALD and adrenal insufficiency.

**Table 1:** Diagnostic investigations supporting the diagnosis of cerebral X-linked adrenoleukodystrophy and primary adrenal insufficiency.

Test/Analyte	Result	Reference Range	Interpretation
Very long chain fatty acid			
C26:0 (nmol/ml)	2.3	≤1.39	Elevated
C22:0 (nmol/ml)	28.5	≤96.3	Normal
C24:0 (nmol/ml)	67.1	≤91.4	Normal
C24:0/C22:0 ratio	2.35	≤1.39	Elevated
C26:0/C22:0 ratio	0.081	≤0.023	Elevated
Synacthen stimulation test			
ACTH (baseline, pmol/L)	243	1.6–13.9—*Markedly elevated*	Markedly elevated
Serum cortisol (baseline, nmol/L)	266	—	
Serum cortisol (60 min post-synacthen, nmol/L)	283	Normal: rise >200 nmol/L or absolute >550 nmol/L	Suboptimal
Absolute cortisol rise (nmol/L)	17	—	Inadequate adrenal response
MRI Brain	Symmetrical T2 hyperintensities involving bilateral frontal periventricular and deep white matter, corticospinal tracts (corona radiata and posterior limb of internal capsule), splenium of the corpus callosum, and cerebellar white matter—*Consistent with early cerebral involvement in X-linked ALD*		Consistent with early cerebral involvement in x-linked ALD

Summary of biochemical and radiological findings in our patient with X-linked adrenoleukodystrophy (X-ALD) with cerebral involvement (cALD). Elevated very long chain fatty acids and abnormal VLCFA ratios support the biochemical diagnosis. Synacthen testing confirmed primary adrenal insufficiency. MRI findings of symmetrical white matter involvement, particularly affecting corticospinal tracts and the corpus callosum, are consistent with early cerebral demyelination typical of cALD.

The family had consented to trio WGS upon the first suspicion of genetic leukodystrophy following imaging before receipt of biochemical metabolic results. Clinical whole genome sequencing (WGS) trio analysis confirmed that the proband was hemizygous for an inherited pathogenic variant *ABCD1*(NM_000033.4):c.521A>G (p.Tyr174Cys) and that this variant was maternally inherited (8). The ABCD1 variant identified in our patient, c.521A>G (p.Tyr174Cys**)**, has been previously reported as pathogenic in multiple databases, with patients having this mutation developing ALD. It is located at exon 1 of the gene and it encodes part of the transmembrane domain (TMD), which plays a critical role in peroxisomal membrane integration and substrate translocation. Functional studies have shown that this specific mutation impairs protein trafficking, and the mutant Tyr174Cys ALD protein fails to localize to peroxisomes, as demonstrated by immunofluorescence assays, and is instead targeted for degradation via the proteasomal pathway (9). Although this variant has been detected in patients with cALD, it is not possible to predict precisely from this genotype which patients will develop cerebral ALD, indicating an environmental or epigenetic contribution to the development of cerebral ALD. However, some mutations result in a truncated ALDP lacking the N-terminus, which allows partial function and correct peroxisomal localization—these mutations have been exclusively associated with adrenomyeloneuropathy (a milder neurological phenotype that presents at an older age) rather than cALD, suggesting that residual ALDP activity may influence phenotype (10).

While WGS identified the *ABCD1* variant confirming the cALD diagnosis, it also incidentally revealed the proband was compound heterozygous for two pathogenic variants in *CFTR*, maternally-inherited *CFTR*[NM_000492.4]:c.1865G>A (p.Gly622Asp) (11), and paternally-inherited *CFTR*[NM_000492.4]:c.1521_1523del (p.Phe508del) (12). This conferred a genetic diagnosis of Cystic Fibrosis. The *CFTR*[NM_000492.4]:c.1521_1523del (p.Phe508del) variant is an established pathogenic variant associated with classical CF (13), and the *CFTR*[NM_000492.4]:c.1865G>A (p.Gly622Asp) variant is associated with atypical or mild CF with variable severity (14, 15). In the CFTR2 database, only 14 patients have the p.Gly622Asp mutation, and the consequences of this mutation vary, with some patients who have another CF-causing variant exhibiting clinical CF and others who do not. Furthermore, this variant has not been identified in Malay or Pakistani populations, indicating there is a genomic knowledge gap in various underrepresented racial groups (16).

**Figure 2:**
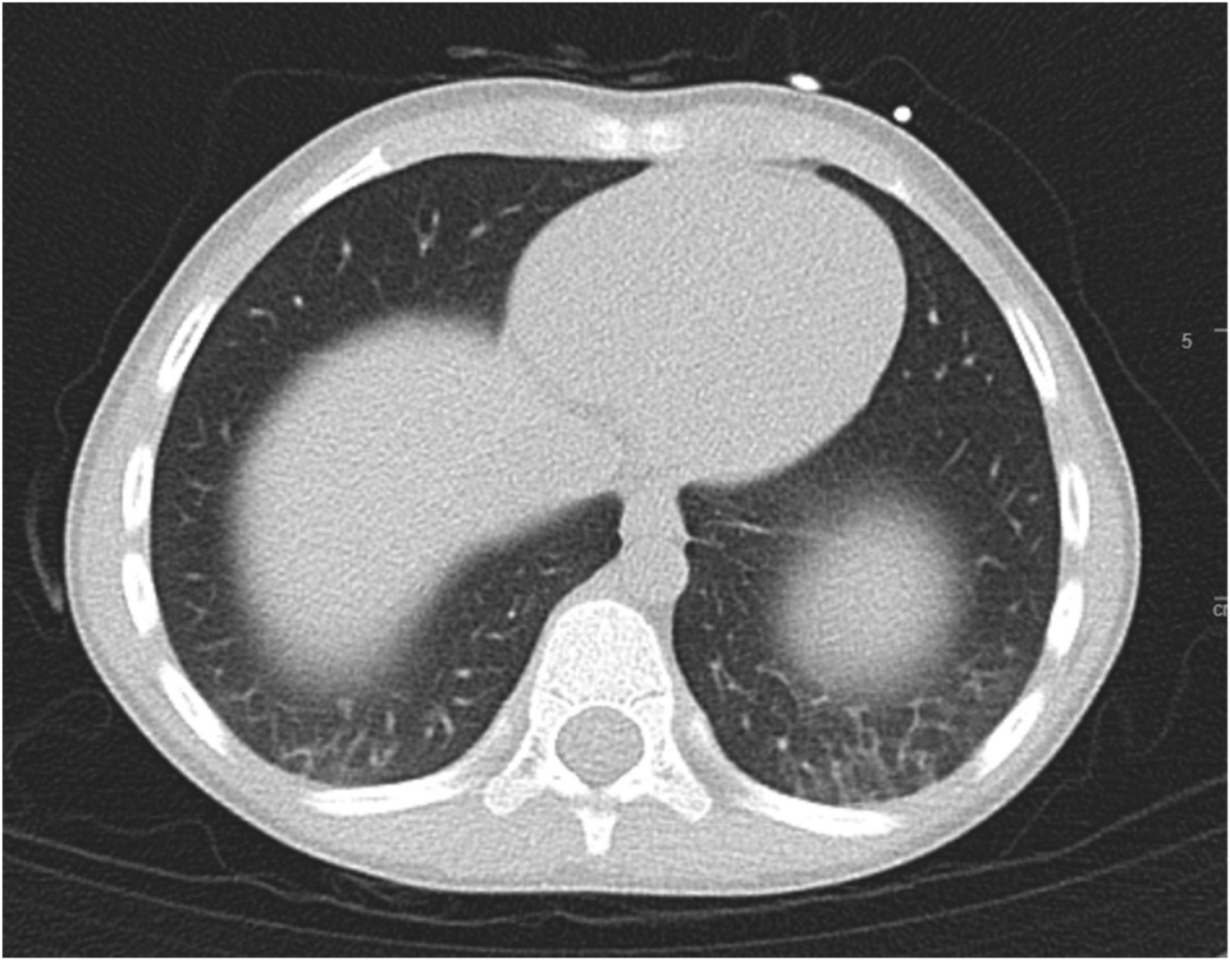
Ct thorax cross-sectional imaging revealing mild bronchiectasis over bilateral bases, more so over the left.

A sweat chloride test was positive at 63 mmol/L (ref >60 mmol/L positive for CF, 30–59 mmol/L intermediate, <29 mmol/L negative) on the right hand and intermediate (48 mmol/L) on the left. A non-contrast computed tomography scan of the thorax revealed mild bronchiectasis near the left lung base without mucus plugging or consolidation (Figure 2).

Unfortunately, he could not cooperate with lung function testing given his age and neurological limitations. Upon diagnosis, he was at the 30th percentile for height (116 cm) and the 40th percentile for weight (21.2 kg), and stool elastase was 500 mcg/g (reference >200 mcg/g, normal), indicating the absence of pancreatic insufficiency and adequate nutrition. The presence of early bronchiectasis on imaging, a clinical feature, but without obvious symptoms, associated with two known pathogenic variants in CFTR and a positive sweat chloride test, were consistent with the diagnosis of early or mild CF (17). Table 2 summarises the supporting results diagnostic of CF.

**Table 2:** Clinical and diagnostic features supporting mild cystic fibrosis.

Parameter	Result	Reference range	Interpretation
Chest CT Scan	Mild bronchiectasis localised near left lung base; no mucus plugging or consolidation	—	**Consistent with CF**
Lung function test	Unable to cooperate	—	—
Sweat chloride test			
Right forearm	63 mmol/L	>60 mmol/L—Pos 30–59 mmol/L—Intermediate <30 mmol/L—Neg	**Positive**
Left forearm	48 mmol/L	>60 mmol/L—Pos 30–59 mmol/L—Intermediate <30 mmol/L—Neg	Intermediate
CFTR Genetic Analysis	2 pathogenic variants	—	**Consistent with CF diagnosis**
Stool elastase	500 µg/g	>200 µg/g	Normal exocrine pancreatic function

Diagnostic features in our patient mild cystic fibrosis. Diagnosis was based on a positive sweat chloride result on one side, the presence of two known pathogenic CFTR variants, and early structural lung changes (mild bronchiectasis) on imaging. Nutritional status and pancreatic function were preserved at diagnosis.

His MRI-based Loes severity score was 7 (range 0–34), suggesting early cerebral disease, which predicted an improved response to HSCT. Urgent HSCT was indicated before neurological progression (18). However, the additional diagnosis of CF altered the patient's risk profile, and pre-HSCT evaluation and consideration for treatment protocol were discussed. The main consideration was to determine how the conditioning regimen would impact his underlying CF. For example, the use of busulfan or radiation may worsen outcomes, causing pulmonary toxicity. However, there was no available literature to guide this decision. Upon reviewing the best available evidence and consulting with an expert in HSCT for cALD, the conditioning protocol for cALD, as advised by the University of Minnesota, was ultimately adopted unmodified (Paul Orchard, personal communication). In cALD, it is critical that busulfan be included in the conditioning regimen despite its potential pulmonary toxicity. This is because busulfan crosses the blood-brain barrier and eliminates central nervous system microglia, allowing donor myeloid engraftment into the central nervous system, thereby arresting the progression of cALD (19). In preparing our patient for HSCT, although he was clinically asymptomatic, the presence of bronchiectasis identified on CT led to the initiation of chest physiotherapy and nebulized hypertonic saline to prevent infectious exacerbation of CF during the period of profound cytopenia post-HSCT.

As no fully matched donors were found in a worldwide donor search, the team proceeded with a haploidentical approach (5/10 HLA match) from the father using CD3/CD45RA-depleted graft technology to mitigate graft-vs.-host disease while enhancing post-transplant infection control (via donor memory T cells add-back). This strategy enabled both rapid donor accessibility and optimal immunologic safety.

Busulfan, cyclophosphamide, fludarabine, and anti-thymocyte globulin (ATG) were administered according to institutional cALD protocols and the University of Minnesota conditioning protocol for cALD (Paul Orchard, personal communication). Donor myeloid engraftment was observed at Day+13 post-HSCT. The patient experienced mild mucositis, a transient febrile episode responsive to broad-spectrum antibiotics, and no severe respiratory decompensation. Liver function tests revealed mild transient transaminitis, possibly from conditioning-related toxicity that resolved without intervention.

Upon discharge, the patient's neurological deficits progressed as expected while awaiting donor myeloid reconstitution in the central nervous system. Clinically, he needed assistance ambulating and was predominantly wheelchair bound shortly after HSCT. He was able to express understanding of simple commands but was not verbal. Additionally, he had slightly decreased tone and power and mild ankle contractures. However, after about 8 months post-HSCT, he gradually became able to walk independently, had normal swallowing function, although his verbal output remained limited. The Loes score 1 year post-HSCT was stable at a score of 14. Outpatient care now includes routine pulmonary monitoring and continued speech/occupational therapies to address preexisting deficits. A paediatric respiratory follow-up is in place to evaluate and treat potential CF-related exacerbations.

## Discussion

**Figure 3:**
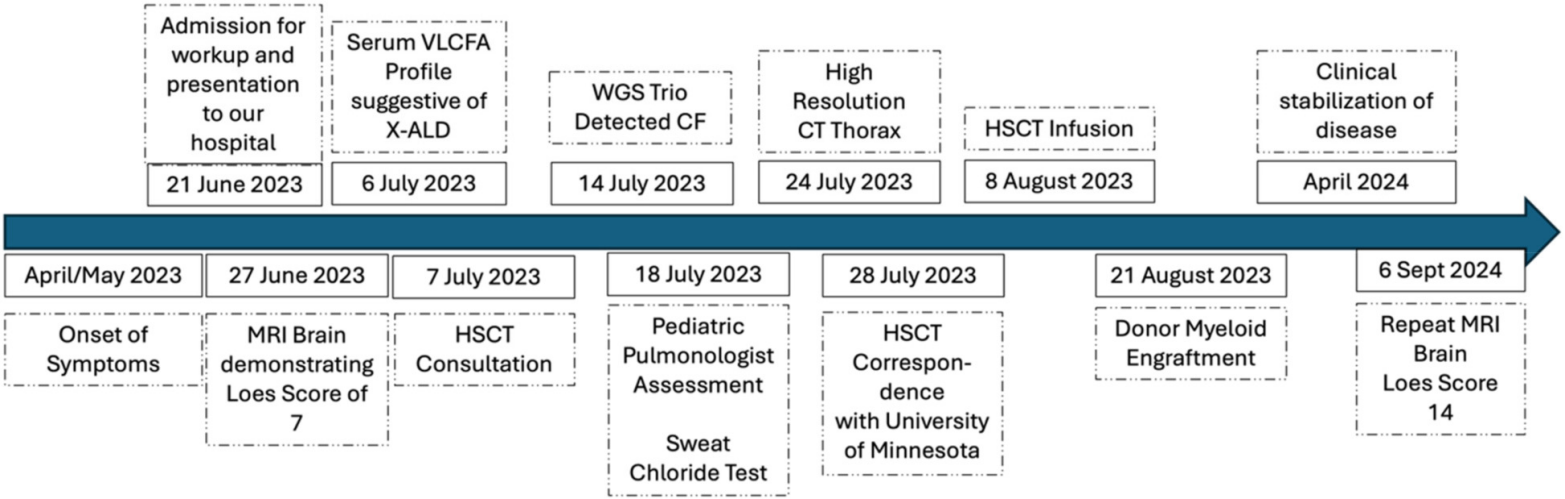
Timeline of key events in the case report.

Comprehensive genetic testing (WES, WGS) is increasingly utilized to personalize haematopoietic stem cell transplantation (HSCT) conditioning regimens, primarily in bone marrow failure syndromes and primary immunodeficiencies. In these patient populations, genetic-guided adjustments may reduce toxicity due to heightened sensitivity associated with specific genetic diagnoses such as Fanconi anaemia, dyskeratosis congenita, or primary immunodeficiencies (7, 20–22). In contrast, our report describes a unique scenario in which WGS performed for cALD uncovered an incidental diagnosis of cystic fibrosis (CF)—a diagnosis unexpected in our population of HSCT patients—which highlighted unique concerns regarding regimen-related toxicity and clinical management, prompting tailored management and follow-up (Figure 3). This demonstrates how modern genomic technologies can uncover hidden comorbidities that alter clinical planning—in this case, establishing a concurrent cystic fibrosis (CF) diagnosis early enabled pre-emptive management of early bronchiectasis and informed the risk associated with conditioning and subsequent follow-up. Although no dose modifications were made to the HSCT conditioning protocol, some institutions have used alternatives to chemotherapy-based conditioning regimens, including total body radiation or total lymphoid radiation, that may exacerbate the underlying CF. Our HSCT team had to balance the use of busulfan in the conditioning regimen, which is known to have pulmonary toxicity rates from 2.5%–8.3%, manifesting as acute pulmonary syndrome, late onset interstitial pneumonitis and fibrosis, and even bronchiolitis obliterans (23). However, this was balanced against the need for busulfan within the conditioning regimen, which has been shown in pre-clinical models to be necessary for central donor myeloid engraftment to halt the progression of cALD. Hence, busulfan-based conditioning is predominantly used throughout the world for cALD (19). Other regimens in the past had used radiation; however, this was eliminated from consideration, as cohorts have reported that irradiation is associated with poorer functional outcomes post-HSCT, and in our patient, would also contribute towards radiation-induced lung injury (24).

The relationship between cALD and CF is currently unknown, and this represents a knowledge gap in our current understanding of dual diagnosis in this context. In the context of HSCT for cALD, CF may modify airway management, antibiotic selection, conditioning regimens, and monitoring for the development of serious complications later. There are no published studies on the consequences of underlying CF in HSCT. Upon review of the literature, there is only one case report of a paediatric patient with CF who underwent HSCT for acute lymphoblastic leukaemia, and his course was complicated by invasive pulmonary fungal infection requiring a lobectomy (25). Regardless, multiple studies have shown that HSCT conditioning, in general, can be associated with pulmonary toxicity. The use of chemotherapy agents, including busulfan, fludarabine, and radiation, can cause acute pulmonary dysfunction and may contribute to late effects (23). Other complications post-HSCT include that of bronchiolitis obliterans—a morbid form of chronic graft vs. host disease (26). Moreover, it has also been demonstrated that HSCT is associated with pulmonary dysfunction in children, with up to 64% of children receiving HSCT having one abnormal lung function parameter post-HSCT (26–28). As such, it is conceivable that patients with underlying CF would be more susceptible to these complications and would need closer monitoring post-HSCT. In our patient, the adoption of CF-specific measures (airway clearance and evaluation) possibly mitigated transplant morbidity. Further, the benefit of early diagnosis of CF is likely to impact his care in terms of late effects follow-up, with more active surveillance of respiratory complications post-HSCT. However, limitations include the brief follow-up period and uncertainty regarding the child's evolving CF phenotype.

There are no guidelines for the routine use of genetic sequencing before HSCT. One large study recommended the routine use of exome sequencing before HSCT but in the context of malignancy to reveal harmful germline variants in disease-predisposing or actionable genes—of which 15.1% were identified in adults and 22.9% in children in a cohort of 432 HSCT patients, they then recommended that the findings underscore the benefit of routine germline genetic testing in HSCT workflows, as many family-donor transplants proceeded without prior genetic diagnosis (29). Meanwhile, for incidental findings, the American College of Medical Genetics and Genomics has generated a list of actionable incidental findings in clinical exome sequencing that primarily focus on cancer predisposition, cardiovascular phenotypes, and some inborn errors of metabolism; however, it is pertinent to note that cystic fibrosis is not included on this list (5).

In conclusion, this child's incidental CF diagnosis influenced peri-transplant decisions and has impacted post-transplant care with more careful surveillance. The care team prevented serious complications by integrating CF-specific measures into the HSCT approach, highlighting how modern genomics and incidental findings can refine the care pathway for patients with complex genetic diseases, such as cALD, undergoing high-stakes procedures like allogeneic HSCT. This suggests that WGS may be beneficially incorporated into the pre-HSCT evaluation for patients receiving allogeneic HSCT to detect clinically significant diseases with actionable consequences, particularly in patients with atypical phenotypic presentations, a history of consanguinity, or ethnically diverse populations, such as in our patient.

### Patient's perspective

From the patient's perspective, the patient's parents shared that reading this case report provided them with a deeper understanding of their son's condition. They expressed that the report helped clarify the clinical and genetic aspects of the diagnosis, allowing them to better grasp the implications for their son's health and future care.

## Data Availability

The original contributions presented in the study are included in the article/Supplementary Material, further inquiries can be directed to the corresponding author.
